# Lower Oncogenic Potential of Human Mesenchymal Stem Cells Derived from Cord Blood Compared to Induced Pluripotent Stem Cells

**Published:** 2015-08-01

**Authors:** T. Foroutan, M. Najmi, N. Kazemi, M. Hasanlou, A. Pedram

**Affiliations:** 1 Department of Animal Biology, Faculty of Biological Sciences, Kharazmi University,; 2 Department of Cell Biology, Faculty of Sciences and New Technologies, Pharmaceutical Sciences Branch of Islamic Azad University,; 3 Department of Molecular Genetic, Faculty of Biological Sciences, Tarbiat Modares University

**Keywords:** Mesenchymal stem cells, Cord blood, OCT4, Sox2, c-Myc; Nanog, lin28

## Abstract

**Background:**

In regenerative medicine, use of each of the mesenchymal stem cells derived from bone marrow, cord blood, and adipose tissue, has several cons and pros. Mesenchymal stem cells derived from cord blood have been considered the best source for precursor transplantation. Direct reprogramming of a somatic cell into induced pluripotent stem cells by over-expression of 6 transcription factors Oct4, Sox2, Klf4, lin28, Nanog, and c-Myc has great potential for regenerative medicine, eliminating the ethical issues of embryonic stem cells and the rejection problems of using non-autologous cells.

**Objective:**

To compare reprogramming and pluripotent markers OCT4, Sox-2, c-Myc, Klf4, Nanog, and lin28 in mesenchymal stem cells derived from cord blood and induced pluripotent stem cells.

**Methods:**

We analyzed the expression level of OCT4, Sox-2, c-Myc, Klf4, Nanog and lin28 genes in human mesenchymal stem cells derived from cord blood and induced pluripotent stem cells by cell culture and RT-PCR.

**Results:**

The expression level of pluripotent genes OCT4 and Sox-2, Nanog and lin28 in mesenchymal stem cells derived from cord blood were significantly higher than those in induced pluripotent stem cells. In contrast to OCT-4A and Sox-2, Nanog and lin28, the expression level of oncogenic factors c-Myc and Klf4 were significantly higher in induced pluripotent stem cells than in mesenchymal stem cells derived from cord blood.

**Conclusion:**

It could be concluded that mesenchymal stem cells derived from human cord blood have lower oncogenic potential compared to induced pluripotent stem cells.

## INTRODUCTION

**Figure 1:**
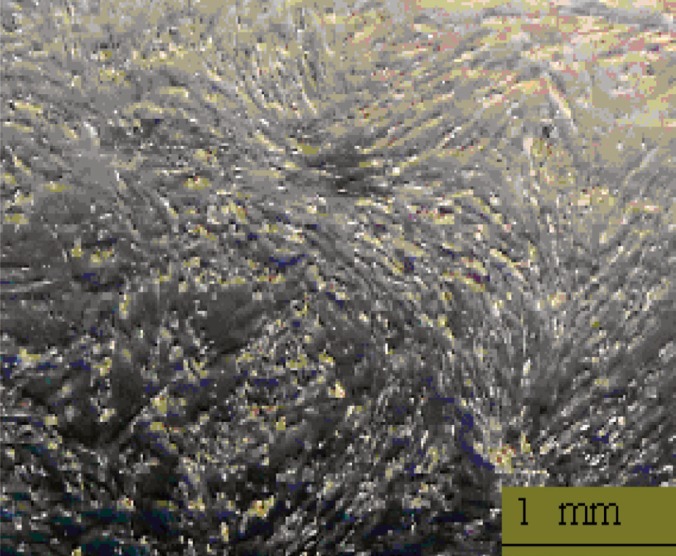
Phase contrast microscopy image of mesenchymal stem cells derived from cord blood in the second passage.

Embryonic stem (ES) cells are pluripotent stem cells and derived from the inner cells mass of embryo. These cells are capable of differentiation into every cell. In contrast to adult stem cells, ES cells can be cultured indefinitely while maintaining their pluripotency [1-3]. Because of ethical concerns associated with generation of ES cells, there is paucity of information on these cells regarding their potential application, particularly in regenerating musculoskeletal tissues. Recently, it has been demonstrated that mouse and human somatic cells can be reprogrammed into an ES cells-like state by introducing combinations of transcription factors. Indeed potentially induced pluripotent stem cells (iPSCs) possess for generating ES cells. On the other hand, adult stem cells are harvested from different tissue sources and variously called multipotent mesenchymal stromal cells or mesenchymal stem cells (MSCs) (Fig 1) [4-8]. MSCs could differentiate into osteoblast, chondroblast, cardiomyocyte, or even cells of nonmesodermal origin including hepatocytes and neurons [9]. Although MSCs are originally isolated from bone marrow, similar populations have been reported in other tissues such as umbilical cord blood and adipose tissue. Adult stem cells have generated great interest because of their potential application in regenerative medicine. In perspective of regenerative medicine, use of each of these sources has advantages and disadvantages. Unlike bone marrow-derived MSCs, adipose tissue-derived stem cells (ADSCs) can be obtained in large quantities at low risks [10]. In addition to being more abundant and easily accessible, the adipose tissue yields far more stem cells than bone marrow on a per gram basis (5000 *vs*. 100-1000) [11]. Indeed adipose tissue provides a readily accessible and rich deposit of stem cells for increasing trend in the liposuction procedure. Biopsy of adipose tissue for extraction of stem cells is also less harsh on patients as compared to procedures involving bone marrow tissue. ADSCs are also a more reliable source for auto-graft transplants as compared to the bone marrow-derived MSCs. These are capable of autologous and allogenic tissue grafting, and there is a minimal immunogenic response to such a procedure [12, 13]. On the other hand bone marrow-derived MSCs require a higher density of cells for the initial culturing (>50,000 cells/cm^2^) than the ADSCs (3000 cells/cm^2^) [13]. The high accessibility of adipose tissue and their remarkable properties make them a suitable candidate for regenerative medicine application. However, adult stem cells have limitations in their application because they cannot be propagated indefinitely in culture; number of these cells also decrease with aging and there is evidence that these cells may exhibit reduced proliferation and differentiation with aging [14-18]. MSCs derived from cord blood have been considered the best source for precursor transplantation [19, 20]. Cord blood stem cells, compared to other adult stem cells, have advantages of having allogeneic properties such as easy access and higher ability to accumulate at the site of tissue damage and immunohistochemical features [21]. Today CD133+ cells isolated from umbilical cord blood is used to treat patients with hematopoietic disorders. Over the last two years, the use of MSCs derived from placenta and umbilical cord blood has increased. In addition to clinical trials, more than 6000 patients treated under compassionate use as isolated clinical cases are described in the literature. For this system, patients with widely different conditions have been treated, from tracheal resections to type I diabetes, as well as skin regeneration and bone reconstructions, both in adult and pediatric patients. Unfortunately, cell therapy with MSCs is not yet a clinical reality. 

Yamanaka and Takahashi managed to reprogram the mouse somatic cell to pluripotent ES cells like by the simple retroviral over-expression of four transcription factors—Oct-4, Sox-2, Klf-4, and c-Myc [22]. Stem cells derived from this process are named iPSCs and closely resemble ES cells since they restore a genome associated with a pluripotent marker expression and fulfill all major biological criteria for pluripotency such as *in vitro* differentiation into cell types of all three germ layers. 

The objective of the present study was comparison of expression of pluripotent genes Oct-4, Sox-2, c-Myc, Klf4, Nanog and lin28 in isolated MSCs from umbilical cord blood as the best source for precursor transplantation and iPSCs. 

## METHODS AND MATERIALS

Cell Culture

MSCs from cord blood:

Human umbilical cord blood was collected after taking the informed consent of the mother using the guidelines approved by the Ethics Committee on the use of human subjects by a standardized procedure using SiRNA containing L-heparin as anticoagulant. After 2:1 dilution with PBS, mononuclear cells were obtained by Ficoll density-gradient centrifugation at 400 g for 25 m [20]. The cells were washed twice in PBS and seeded at a density of 1×10^6^ cells/cm^2^. Growth of adherent cells was initiated in myelocult medium (Stem Cell Technologies) with dexamethasone (1027 M; Sigma-Aldrich), penicillin (100 U/mL; Gibco), streptomycin (0.1 mg/mL; Gibco), and glutamine (2 mM; Gibco). Nonadherent cells were removed after 72 h, and the adherent cells were fed weekly with culture medium. Expansion of the cells was performed in Mesencult basal medium (M3; StemCell Technologies) with additive stimulatory supplements. iPSCs used in this study were purchased from Royan Institute.

Quantitative real-time PCR:

RNA of treated and non-treated MSCs, cord blood- and adipose tissue-derived stem cells were extracted using Trizol reagent (Invitrogen) according to the manufacturer’s protocol. RNA was analyzed with quantitative real-time PCR (qPCR). Melting curve analyses and PCR product sequencing were performed to verify primer specificities. RT-PCR was repeated at least three times using the following conditions: Each of the reaction mixtures contained 10 µL of SYBR green master mix (Applied Biosystems), 5 pM each of forward and reverse primers, and 5 µL of 100 times diluted cDNA. The primer sequences used for qPCR are shown in Table 1.

**Table 1:** Primer pairs designed for light cycler RT-PCR

Oct4	forward: 5’-CGCAAGCCCTCATTTCAC-3’ reverse: 5’-CATCACCTCCACCACCT-3’
Sox-2	forward: 5’-TGCTGCCTCTTTAAGACTAGGAC-3’ reverse: 5’-CCTGGGGCTCAAACTTCTCT-3’
c-Myc	forward: 5’-CACCAGCAGCGACTCTGA-3’ reverse: 5’-GATCCAGACTCTGACCTTTTGC-3’
Nanog	forward: 5’-AGATGCCTCACACGGAGACT-3’ reverse: 5’-TTTGCGACACTCTTCTCTGC-3’
Klf4	forward: 5’-GGGAGAAGACACTGCGTCA-3’ reverse: 5’-GGAAGCACTGGGGGAAGT-3’
lin28	forward: 5’-AGGCAGTGGAGTTCACCTTTAAGA-3’ reverse: 5’-AGCTTGCATTCCTTGGCATGATGA-3’
GAPDH	forward: 5’- ATGGGGAAGGTGAAGGTCG-3’ reverse: 5’-GGGGTCATTGATGGCAACAATA-3’

## RESULTS

Several studies have reported that over-expression of OCT-4, Klf-4, Sox-2, and c-Myc is sufficient to induce cellular reprogramming. In the present study we compared the expression levels of these genes in human MSCs derived from cord blood and iPSCs. All types of MSCs used in the present study were in the third passage as confirmed by adult stem cell markers such as CD34, CD45, CD90, CD105, CD73, and CD38. 

**Figure 2:**
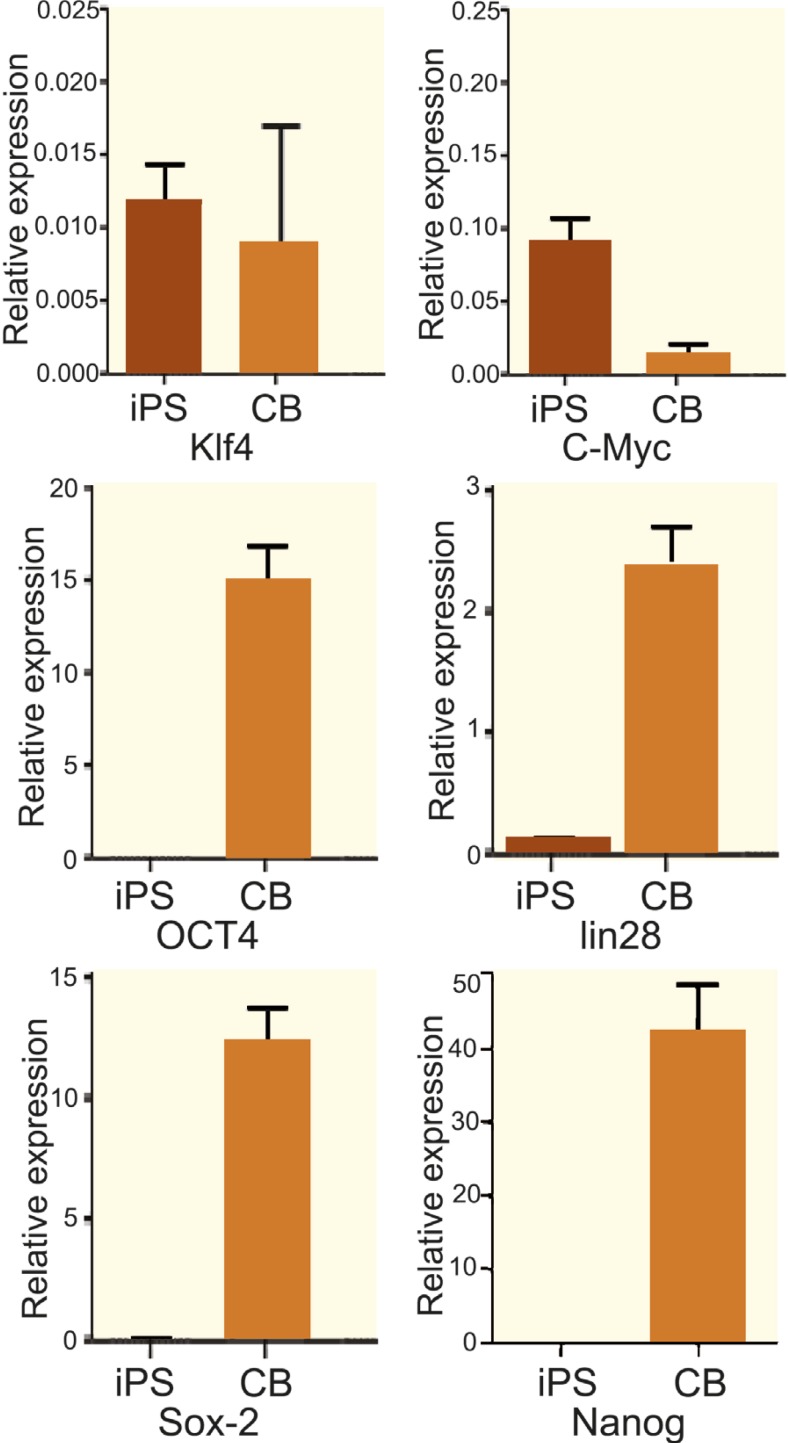
Comperative real-time PCR analysis of Klf4, c-Myc, OCT4, lin28, Sox-2 and Nanog genes expression in MSCs derived from iPSCs and cord blood (CB). Expression of c-Myc and Klf4 oncogenes in MSCs derived from CB is significantly lower than that in iPSCs. Also the expression of OCT4, lin28, Sox-2, and Nanog, pluripotent genes in MSCs derived fromCB is significantly (p<0.05) higher than that in iPSCs

Our data showed a significantly higher level of OCT4, Sox-2, Nanog, and lin28 expression in MSCs derived from cord blood compare to iPSCs (Fig 2a). In contrast to OCT-4, Sox-2, lin28, and Nanog, the expression level of oncogenic factors c-Myc and Klf4 were significantly higher in iPSCs than cord blood (Fig 2b).

## DISCUSSION

Direct reprogramming of somatic cells into iPSCs by transcription factors (OCT4, Sox2, Klf4, c-Myc, Nanog, and lin28 ) has great potential for tissue-specific regenerative therapies, eliminating the ethical issues surrounding the use of embryonic stem cells and the rejection problems of using non-autologous cells [24]. The present study showed that the expression of pluripotent genes and reprogramming factors OCT-4 and Sox-2, Nanog, and lin28 in MSCs derived from cord blood were higher than iPSCs significantly. OCT-4 and Sox-2 are widely accepted markers for embryonic stem cells as well as the reprogramming factors [25]. OCT4 expression has already been reported in several adult somatic cells [25]. Reports of OCT-4 expression in adult human differentiated cells then challenged its role as a pure stem cell marker [23]. Wagnera believed that the expression of OCT4 in adult stem cells is less certain [27]. Tai and colleagues reported that OCT4 expression in somatic cells is restricted to small populations of multipotent cells with high self-renewal capacity, namely the adult stem cells in normal tissues [28]. Recently, researchers succeeded in inducting pluripotent stem cells from primary human fibroblasts with only OCT4 and Sox-2 factors [27, 29]. In relevance to Ratajczak and colleagues suggestion, OCT4 is an embryonic transcription factor that incurred at low concentrations within somatic cells [30]. The present experiment has shown that the expression of OCT4 transcriptional factor was significantly higher in MSCs derived from cord blood than iPSCs. In 2008, Guiting, *et al*, reported that expression of this gene in adipose-derived stem cells is very low [31]. In contrast to the findings of other researchers, Izadpanah, *et al*, concluded that OCT-4 is not specific to embryonic stem cells [28]. In accord to Izadpanah and colleagues’ findings, our results showed OCT4 is not specific to embryonic stem cells [28]. One possible explanation could be that MSCs derived from cord blood have some properties of embryonic stem cells while being considered adult stem cells. Our data showed that cord blood stem cells express the main pluripotent stem cells markers, OCT4 and Sox-2, lin28 and Nanog iPSCs. This might confer a more reprogrammable state for MSCs from cord blood. The other possible explanation could be based on Bhartia hypothesis. He and colleagues in 2012 reported that the true stem cells in adult body tissues are the very small embryonic-like stem cells (VSELs), whereas the MSCs are actually progenitor stem cells that arise by asymmetric cell division of VSELs [32]. According to this hypothesis, cord blood contains VSELs that are possibly lost during cord blood banking, therefore adult autologous stem cell trials efficacy is low. It seems that cord blood-derived stem cells have a higher number of VSELs compared to iPSCs. In view of our data, it can be concluded that cord blood-derived stem cells can be considered a source of pluripotent stem cells that have a more regenerative potential than bone marrow- and adipose-derived MSCs. In 2009 it has been reported that Klf4 interacts directly with OCT4 and Sox2 when expressed at levels sufficient to induce iPSCs [33]. 

Our findings revealed that the expression level of c-Myc and Klf4 in cord blood stem cells were significantly lower than iPSCs. It is well known that the reprogramming factors Klf4 and c-Myc are more oncogene than other pluripotent genes [34-37]. It means that omission of c-Myc and Klf4 from the reprogramming process is important since reactivation of the c-Myc virus in MSCs derived from AD can endogenously express high levels of c-Myc; therefore, we propose that these cells can be reprogrammed into iPSCs merely by OCT-4 expression [37, 39]. The increased expression of c-Myc is observed in 70% of human tumors because it is a notorious ontogeny in human cancers [40]. Tsai and colleagues reported that over expression of only OCT4 and Klf4 genes were sufficient to induce reprogramming without exogenous or endogenous c-Myc [24]. Before we showed that MSCs derived from AD endogenously express high levels of c-Myc and Klf4, we proposed that these cells can be reprogrammed into iPSCs merely by OCT4 expression [41]. It seems that MSCs derived from cord blood are less oncogene than iPSCs. Gletting, *et al,* found that adipocyte assists in suppressing human hematopoietic stem cells differentiation and aid in prolonging their survival *in vivo* [42]. It can be proposed that OCT4, Sox-2, Nanog, and lin28 act as reprogramming genes and c-Myc and Klf4 function by aiding in the activation of pluripotent genes. It may be concluded, due to the tumorogenic properties of Klf4 and c-Myc factor, that any increase in their expression in ADSCs could be catastrophic.

Based on the present data, considering the high expression of OCT4, Sox-2, Nanog, and lin28 embryonic stem cell marker, MSCs derived from cord blood are proposed as a more appropriate candidate for cellular therapy compared to MSCs derived from the bone marrow and AD. 

In conclusion, MSCs derived from cord blood are more appropriate candidates for cell therapy compared to iPSCs. It could be concluded that MSCs derived from human cord blood have lower oncogenic potential compared to iPSCs.
